# Effects of multiple transcranial magnetic stimulation sessions on pain relief in patients with chronic neuropathic pain: A French cohort study in real‐world clinical practice

**DOI:** 10.1002/ejp.4763

**Published:** 2024-12-10

**Authors:** Joy Thomas, Camille Fauchon, Nicolas Oriol, François Vassal, Christelle Créac'h, Charles Quesada, Roland Peyron

**Affiliations:** ^1^ Inserm U1028 Neuropain Université Jean‐Monnet, F‐42023, Saint‐Etienne and Centre de Recherche en Neurosciences de Lyon (CRNL) UMR5292 Saint‐Etienne et Lyon France; ^2^ Centre Stéphanois de la Douleur et Département de Neurologie Centre Hospitalier Régional Universitaire de Saint‐Etienne Saint‐Etienne France; ^3^ Service de Neurochirurgie Centre Hospitalier Régional Universitaire de Saint‐Etienne Saint‐Etienne France

## Abstract

**Background:**

Current clinical trials indicate that repetitive transcranial magnetic stimulation (rTMS) is effective in reducing drug‐resistant neuropathic pain (NP). However, there is a lack of studies evaluating the long‐term feasibility and clinical efficacy of rTMS in large patient cohorts in real‐world conditions.

**Methods:**

In this retrospective cohort study, we analysed 12 years of clinical data to assess the long‐term analgesic effects of 20 Hz rTMS over the primary motor cortex in patients with NP. Subgroup analyses were conducted to identify predictive factors and assess the potential role of epidural motor cortex stimulation (eMCS) as a sustained solution.

**Results:**

In total, 193 patients completed test period of 4 rTMS sessions and 42% of them reported a pain relief (PR) greater than 30%, with concurrent improvement in their most disabling symptom. Iterative rTMS sessions maintained analgesic effects over 10 years in certain patients identified as responders (≥10% PR) without adverse effects. Success probability was higher in patients with central NP compared to peripheral NP (OR = 2.03[1.04;4.00]), and among those with central post‐stroke pain, this probability was higher in ischemic versus hemorrhagic strokes (OR = 3.36[1.17;10.05]). PR obtained with iterative rTMS sessions was an excellent predictor of eMCS efficacy.

**Conclusions:**

While rTMS shows promise as a therapeutic option for some patients with drug‐resistant NP, it does not benefit all patients. Efficacy varies by NP aetiology, aiding patient selection. For responders, eMCS may offer a permanent solution. These findings support a tailored approach to rTMS in NP management, while recognizing both its potential and limitations across diverse patient profiles.

**Significance Statement:**

Multiple rTMS sessions demonstrate long‐term efficacy and safety in treating drug‐resistant neuropathic pain. Extending session numbers for the test period can enhance responder identification, especially in patients with initial low pain relief. This identification refines patient selection for neurosurgery, reducing non‐responders. Central neuropathic pain shows higher success rates than peripheral. For post‐stroke central pain, patients with ischemic stroke are more likely to respond than those with hemorrhagic stroke. These results support integrating rTMS into clinical practice for managing neuropathic pain.

## 1 INTRODUCTION

Neuropathic pain (NP) is pain that arises as a direct consequence of a lesion or disease of the somatosensory nervous system (Raja et al., 2020). Lesions are located at the peripheral or the central level. Prevalence of neuropathic pain is estimated to 7%–10% in the general population worldwide (van Hecke et al., 2014), from 8% after strokes (Andersen et al., 1995; Demasles et al., 2008; Klit et al., 2009), up to 51% in multiple sclerosis (Moisset et al., 2013) and 53% after spinal cord injuries (Burke et al., 2017). Neuropathic pain is rarely reversible, intense up to a suicide level and is generally refractory to a large panel of drugs (Evoy et al., 2017; Moisset, Pereira, et al., 2020), including opioids (Moisset et al., 2022). Only 30%–40% of them have more than 50% of pain relief (Finnerup et al., 2015). Alternative—nonpharmacological—interventions using neuromodulation techniques have been proposed (Garcia‐Larrea & Quesada, 2022). Among them, repetitive transcranial magnetic stimulation (rTMS) applied over the primary motor cortex (M1) is the neuromodulation technique with the highest level of evidence, in the third‐line treatment, to alleviate chronic neuropathic pain (Moisset, Bouhassira, et al., 2020). This non‐invasive technique has been derived from the original invasive technique of electrical motor cortex stimulation (eMCS) (Tsubokawa et al., 1991). eMCS is a neurosurgical technique first proposed as an empirical method to alleviate pain (Gatzinsky et al., 2021). If rTMS was only used to predict the effect of the eMCS at the beginning (André‐Obadia et al., 2014), since a decade, the rTMS treatment has been increasingly used in clinical protocols, with repeated sessions of high‐frequency stimulations (>10 Hz). A number of randomized controlled trials concluded a statistical superiority of motor cortex rTMS relative to placebo to improve drug‐resistant central (Quesada et al., 2020; Zhao et al., 2020, 2021) and peripheral (Attal et al., 2021; Hosomi et al., 2020) neuropathic pain (CNP, PNP).

However, according to these studies, the efficacy of rTMS treatment remains incomplete (50% of patients do not benefit from the technique) and unreliable (O'Connell et al., 2018). This is likely the result of strong heterogeneity of patients' clinical characteristics, but there is no concrete information on predictors of the success of rTMS treatment. Moreover, in previous studies the sample size was small, the patient profile was highly selective, and information on long‐term effects was not reported. Therefore, it is essential to assess the efficacy and safety of this technique in real‐world settings and identify individual factors that could influence the probability of rTMS efficacy on pain in long term (Baron et al., 2022). Here, we present the long‐term outcomes of our 12 years' experience of 20 Hz rTMS in a cohort of 213 patients with—central or peripheral—neuropathic pain. The aims of this retrospective French cohort study in real‐world clinical practice were to assess a large population of patients the efficacy and safety of rTMS over several years, to identify predictive factors and to determine how to achieve permanent solutions with eMCS.

## 2 METHODS

### 2.1 Overview

The current study, used data from 213 patients who had received iterative rTMS sessions as part of maintenance therapy for neuropathic pain in clinical practice, conducted at one site, the neurology and pain department of the University Hospital of Saint‐Etienne between October 2010 and March 2022. Among them, 25 patients had received epidural motor cortex stimulation (eMCS) and outcomes data were extracted from the neurosurgery department at the same University hospital between October 2010 and June 2023.

### 2.2 Participants

The individuals included were drug‐refractory patients who suffered from typical clinical symptoms of neuropathic pain evaluated by an expert neurologist (Baron et al., 2010) and lasting for at least 6 months with a moderate to severe intensity (numerical rating scale, NRS >4/10). Drug‐refractory refers to inadequate relief or intolerance to gabapentinoids, tricyclic antidepressants and SNRIs, in accordance with French recommendations for the treatment of neuropathic pain (Moisset et al., 2021) with the addition of clonazepam in cases of pain‐related insomnia. Lesion‐causing NP was either directly documented (magnetic resonance imaging (MRI), electromyography) or, if no lesion was documented, neurophysiological investigations (laser or somatosensory evoked potentials, or quantitative sensory testing) showed sensory abnormalities in the pain territories. The individuals with severe motor deficits (i.e. para or tetraplegic), ferromagnetic implants (i.e. cochlear implants), drug‐resistant or active epilepsy, inability to complete self‐questionnaire in French, pacemaker (not totally justified), pregnancy, ongoing depression or personality disorders based on clinical assessment from an expert neurologist, were excluded from this therapy. If necessary, special advice could be obtained from a psychiatrist. Only individuals who received at least four rTMS sessions during the study period were analysed, as previous studies recommend this threshold as the minimum number of sessions necessary to assess the effectiveness of the rTMS treatment (Pommier et al., 2016; Quesada et al., 2018).

For all individuals, we collected demographical data (age, sex) and clinical determinants of pain (level of lesion, aetiology, duration of symptoms, intensity (with NRS/10) laterality and topography of pain). All patients were classified as suffering from central, peripheral or mixed pain. Central pain conditions were categorized on its neuroanatomical origins (cortical, lenticular, thalamic, brainstem or spinal cord) and its aetiology, especially for central post‐stroke pain (ischemic or hemorrhagic).

### 2.3 Neurostimulation interventions

rTMS treatment: TMS stimuli were delivered using a MagPro stimulator (Magventure®) through a figure‐of‐eight coil, targeting the primary motor cortex contralateral to the painful area, or the left side in patients with bilateral pain. Target was marked on each patient's individual MRI (1×1×1 mm 3D‐T1‐weighted image) and a robotized arm (Smartmove, ANT) coupled to a neuronavigation system (Visor2, ANT) was used to perform standardized targeting over repeated sessions and real‐time maintenance of the coil over the target for the entire duration of each session (27 min). The same equipment was used throughout the entire period. Patients were stimulated at a frequency of 20 Hz, power (intensity) level of 80% of motor threshold (MT), 20 trains of 80 pulses per train, 84 s intertrain‐interval and 1600 pulses per session. The effect of rTMS was assessed after a test period made of four consecutive sessions (Nuti et al., 2005; Pommier et al., 2016), separated from each other by 2–3 weeks interval. After this period, patients were classified as rTMS responders or non‐responders, if their self‐reported pain relief was higher or lower than 10%, respectively. Additional sessions were proposed to validate treatment failure (i.e. non‐responders). Patients with pain relief ≥10% were free to either stop or continue a maintenance therapy. If they chose to continue, they could adapt the time interval between rTMS sessions according to their individual duration of the analgesic effect. During the follow‐up, patients were given the option to switch from iterative rTMS sessions to eMCS surgery.

eMCS surgery: One or two electrodes (Resume, Medtronic) with four stimulating contacts were placed over the dura through a fronto‐parietal craniotomy under general anaesthesia. The stimulation electrode was then placed over the motor cortex representation corresponding to the painful area and connected to a subcutaneously implanted stimulator (Synergy or Prime–advanced Medtronic). Based on previous experience (Nuti et al., 2005), the stimulation parameters were adapted in the postoperative stage to optimize the analgesic effects within a range of frequency between 25 and 50 Hz, intensity 1.5–4.5 *V* (always under the threshold of motor response and/or paresthesia), pulse width 60 μs and stimulation cycle ‘on’ for 1 h and ‘off’ for 2 h (Nuti et al., 2005).

### 2.4 Clinical assessment

For each rTMS session, a nurse systematically assessed pain outcomes through a standardized questionnaire. Additionally, patients were systematically questioned on the presence of any side effects at each rTMS session. They were asked if they had noticed any unusual symptoms after stimulation. The same outcomes were used to evaluate eMCS, which is systematically performed by the neurosurgeon approximately every 6 months after the surgery. For this intervention, only the first and last available visits were analysed. Regarding rTMS follow‐up, we picked two landmarks: 4th and 15th sessions. The 4th session is a step for the categorization of responders and non‐responders and the 15th session is a further step for long‐term follow‐up.

The main criterion was the self‐reported pain relief (PR) on a continuous scale from 0% to 100% (0% = no pain relief, 100% = full pain relief) compared with his/her pain intensity prior to the start of rTMS. This pain relief score has been previously validated to measure changes in pain in long‐term follow‐up studies of rTMS and eMCS and is strongly correlated with pain intensity assessed using more conventional measures like the VAS (Nuti et al., 2005; Quesada et al., 2018). Other criteria collected were: (1) the duration of pain relief (DPR) (i.e. the number of days during which patients experienced pain relief) and (2) the neuropathic pain symptom inventory (NPSI). NPSI questionnaire quantifies the mean intensity of 10 neuropathic symptoms and their combination into 5 distinct dimensions (burning, deep pain, paroxysmal pain, evoked pain and paresthesia/dysesthesia) during the last 24 h on a 11‐point (0–10) numerical scale. The NPSI has the advantage to be adapted and validated for the follow‐up of neuropathic pain (Bouhassira et al., 2004) and to provide a non‐specific global score (gNPSI) and sub‐score assessing specifically the most painful component described by patients, among the 5 dimensions (ssNPSI) (Nagoshi et al., 2016; Williams et al., 2000).

The percentage of PR after the test period was used for the categorization of patients into four groups: non‐responders (PR <10%), poor responders (PR between 10% and 39%), good responders (PR between 40% and 69%) and excellent responders (PR ≥70%) (Nuti et al., 2005; Quesada et al., 2018). We also applied a traditional efficacy threshold range, categorizing patients as responders if they reported a percentage of PR ≥30%, or ≥50% after the test period (Dworkin et al., 2005).

### 2.5 Data analysis

Data were summarized using standard descriptive statistics in R version 2022.07.2 statistical software. Quantitative variables were expressed as mean ± standard deviation (sd) and qualitative variables were expressed as percentage of incidence in the sample.

The interest of using a 10% permissive threshold was assessed by comparing follow‐up and rTMS efficacy among patients reporting PR of 10%–29% and 30%–49% after the test period. Differences were evaluated using appropriate statistical tests in compliance with requirements and assumptions; independent Student *t* or Wilcoxon tests for continuous variables and *X* ^2^ or Fisher tests for categorical variables. We used separate linear mixed models (LMMs) to assess the association between sessions (first four sessions) and continuous efficacy measures (PR, DPR, ssNPSI, gNPSI) through regression coefficients (*b*[*IC95*]). The influence of covariates (status after test period and level of lesion) on the variables of interest was tested by adding them in LMMs. After the test period, the evolution of efficacy, treatment and patient outcomes were described for responders up to the endpoint (March 2022). For these analyses, it is important to note that, as the duration of follow‐up was different for each patient, 34 patients were still receiving maintenance therapy at the endpoint. For 25 patients who had surgery, Spearman's rank‐order test was used to analyse the correlation between the PR reported at the first evaluation after surgery and at the last session of rTMS. Differences in PR between the two procedures were compared using a paired Wilcoxon signed rank test. Finally, changes of PR and DPR at the fourth session between CNP and PNP were compared using Wilcoxon signed rank test and the measure of the association between the level of lesion and the status after the test period was expressed with Odds Ratio (OR) and 95% confidence intervals and tested with Fisher's exact test. Statistical significance was set to *p* < 0.05.

## 3 RESULTS

### 3.1 Sample characteristics

**FIGURE 1:**
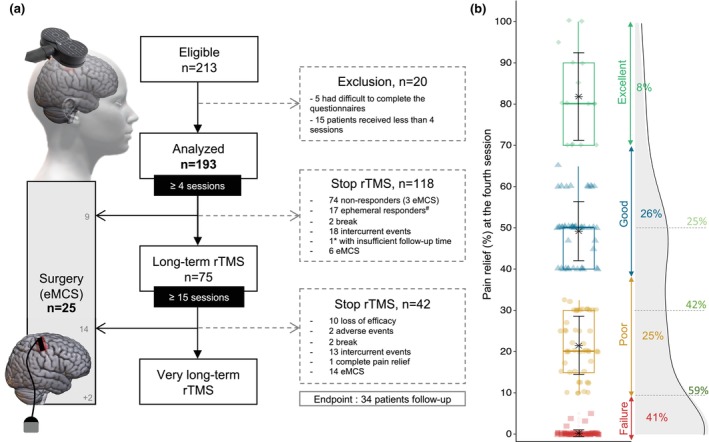
(a) Flow chart of patients included in the analysis. Note two time points: At 4th and 15th sessions. The 4th session is a step for categorization and the 15th session is a further step for long‐term follow‐up. At the endpoint (March 2022) 34 patients were still undergoing maintenance therapy with rTMS (among them, only 1*patient had received less than 15 sessions). Among them, 2 patients received surgery after the rTMS endpoint because the follow‐up for surgery was extended until June 2023. Ephemeral responders^#^: Responders who discontinued rTMS session due to loss of efficacy before the 15th session. (b) Distribution of percentage of pain relief in whole population after the 4th session: Failure (0%–9%, red square), Poor result (10%–39%, yellow dot), Good result (40%–69%, blue triangle), Excellent result (≥70%, green diamond). For each group: Boxplot, mean with error bar in black and frequency of patient. On the right, density diagram of percentage of PR with the evolution of the responder rate according to the permissive ≥10% pain relief as well as more stringent thresholds (≥30% and ≥50% PR): Respectively 59%, 42%, 25%.

In total, 213 patients were treated, for a total of 4228 rTMS sessions delivered and no adverse events were reported except for five patients who experienced reversible post‐stimulation headaches or transient pain enhancement. Five patients were excluded due to challenges in accurately assessing their pain relief either because of language barriers or psychiatric disorders identified too late. An additional, 7% (15 patients) of patients were classified as ‘protocol violations’ because they discontinued treatment before reaching the four‐session threshold. Reasons for these early discontinuations included mostly personal or professional circumstances, as well as, for two patients, tolerance issues encountered early in the study approximately 10 years ago. The final cohort consisted of 193 patients who completed the test period of 4 consecutive sessions and were included in the subsequent analysis (see flowchart in Figure 1a). As shown in Table 1, individuals were 56.2 (±13.6 [min 18; max 88]) years old, and 53% of them were men. Before starting rTMS, pain intensity was 6.3/10 (±2.1) and lasted for 6.4 (±6.9 [min 0.5; max 46.3]) years. In total, 146 patients (70%) had central NP and 46 had peripheral NP. One single patient had two lesions—one central and one peripheral—each one being susceptible to lead to neuropathic pain. See Table S1 for details regarding the characteristics and outcomes of rTMS for each patient.

**TABLE 1:** Baseline characteristics of the 193 patients with drug‐resistant neuropathic pain.

Variables	Values
Age, years (mean ± SD)	56.2 ± 13.6
Male sex, % (*n*)	52.8 (102)
Pain duration, years (mean ± SD)	6.4 (±6.9)
Pain laterality, % (*n*)	
Bilateral	6.2 (12)
Right	44.6 (86)
Left	49.2 (95)
Pain topography, % (*n*)	
Hemibody	47.2 (91)
Lower limb	20.7 (40)
Upper limb	18.7 (36)
Face/Neck	7.3 (14)
Trunk	3.1 (6)
Others	3.1 (6)
Level of lesion, % (*n*)	
Central	75.7 (146)
Spinal cord	30.8 (45)
Brainstem	11.0 (16)
Thalamus	30.1 (44)
Lenticular	6.8 (10)
Cortex	18.5 (27)
Others	2.7 (4)
Peripheral	23.8 (46)
Phantom limb	6.5 (3)
Cranial	23.9 (11)
Radicular	2.2 (1)
Plexus	26.1 (12)
Nerve trunk	23.9 (11)
Sensory fibres	17.4 (8)
Mixed	0.5 (1)
Aetiology, % (*n*)	
Central, *n* = 146	
Post‐stroke pain	57.5 (84)
Myelopathy	6.2 (9)
Multiple sclerosis	5.5 (8)
Traumatic/Surgical nerve	4.1 (6)
Syrinx	4.1 (6)
Oncology	3.4 (5)
Infarct	3.4 (5)
Others	6.8 (10)
Not found	8.9 (13)
Peripheral, *n* = 46	
Traumatic/Surgical nerve	41.3 (19)
Trigeminal neuralgia	17.4 (8)
Sensory polyneuropathy	13.0 (6)
Oncology	8.7 (4)
Postherpetic neuralgia	6.5 (3)
Others	13.0 (6)
Pain intensity (NRS), /10 (mean ± SD)	6.3 ± 2.1
Global score NPSI, /100 (mean ± SD)	36.6 ± 18.0
Superficial burning pain, /10 (mean ± SD)	4.2 ± 3.5
Deep pain, /10 (mean ± SD)	3.3 ± 2.9
Paroxysmal pain, /10 (mean ± SD)	2.8 ± 2.8
Evoked pain, /10 (mean ± SD)	3.4 ± 3.0
Paresthesia/dysesthesia, /10 (mean ± SD)	4.6 ± 2.9
Sub‐score NPSI the most painful component, % (*n*)	
Superficial burning pain	38.0 (41)
Deep pain	13.9 (15)
Paroxysmal pain	5.6 (6)
Evoked pain	13.9 (15)
Paresthesia/dysesthesia	28.7 (31)

### 3.2 Categorization and test period

After the test period, the rTMS efficacy was rated as excellent for 8% of patients, good for 26%, poor for 25% and failure for 41% (Figure 1b). So, 113 patients (59%) were categorized as responders considering a permissive threshold (≥10% PR), with an average PR of 42.1% (±22.1) (Figure 1). Considering less permissive thresholds, 78 patients (42%) and 49 patients (25%) reached PR ≥30% and ≥50% respectively (Figure 1b). No significant differences were observed between patients reporting a PR of 10%–29% (*n* = 32) and those reporting a PR of 30%–49% (*n* = 32), in terms of the frequency of discontinuation due to lack of efficacy after the 4th session (26% vs. 24%, *p* = 0.56), the number of rTMS sessions received (28.6 ± 33.1 vs. 32.8 ± 31.4, *p* = 0.70) and the PR reported at the 15th session (31.9 ± 22.4 vs. 46.5 ± 27.5, *p* = 0.08), as well as at the 30th session (35.9 ± 17.9 vs. 42.7 ± 20.1, *p* = 0.30) (see Figure S1 and Table S2 for further information). 26% of patients (*n* = 9) with 10%–29% PR at the 4th session, reported PR more than 30% at the 15th session.

In the whole population (*n* = 193), significant improvements of the percentage and duration of pain relief, and the sub‐score NPSI were observed in LMMs, during the test period: increase of PR (*b* = 4.55[3.09;6.01]) and DPR (*b* = 1.21[0.77;1.64]) decrease of ssNPSI (*b* = −0.28[−0.41; −0.15]). No significant change was observed for global NPSI (*b* = 0.07[−0.62;0.76], Table 2).

**TABLE 2:** Evolution of percentage and duration of pain relief, global NPSI and sub‐score NPSI at baseline and during the first four sessions (mean ± SD).

	Baseline	Session 1	Session 2	Session 3	Session 4	*b*
Pain relief (%)						
Whole population	–	12.0 ± 22.9	16.0 ± 23.3	21.6 ± 27.4	27.2 ± 28.0	**4.55** [**3.09;6.01**]*
Responders	–	19.2 ± 26.6	25.6 ± 25.3	35.6 ± 27.8	41.4 ± 24.6	**7.72** [**5.58;9.85**]*
Non‐responders	–	6.2 ± 18.0	6.8 ± 16.3	7.1 ± 18.2	8.7 ± 19.6	–
Duration of pain relief (*d*)						
Whole population	–	3.2 ± 6.6	4.8 ± 7.0	6.6 ± 8.8	7.2 ± 7.7	**1.21** [**0.77;1.64**]*
Responders	–	5.3 ± 8.0	8.1 ± 7.7	10.4 ± 9.0	11.0 ± 7.1	**1.93** [**1.26;2.61**]*
Non‐responders	–	1.7 ± 6.4	1.7 ± 5.0	2.1 ± 5.5	2.1 ± 4.5	–
Global NPSI (/100)						
Whole population	36.3 ± 17.7	38.8 ± 20.9	37.6 ± 20.9	38.1 ± 22.4	36.2 ± 22.5	+0.07 [−0.62;0.76]
Responders	37.0 ± 18.4	37.9 ± 22.5	35.2 ± 21.5	35.0 ± 22.7	32.3 ± 22.0	**−1.25** [**−2.08;‐0.41**]*
Non‐responders	36.0 ± 16.8	38.6 ± 18.1	40.2 ± 19.7	40.7 ± 21.4	40.4 ± 22.9	–
Sub‐score NPSI (/10)						
Whole population	6.9 ± 2.1	6.6 ± 2.4	6.2 ± 2.9	6.0 ± 2.7	5.9 ± 2.6	**−0.28** [**−0.41;−0.15**]*
Responders	6.0 ± 2.2	6.3 ± 2.7	5.7 ± 3.1	5.4 ± 2.8	5.2 ± 2.6	**−0.41** [**−0.59;−0.24**]*
Non‐responders	6.9 ± 1.9	7.1 ± 1.8	6.7 ± 2.3	6.4 ± 2.5	6.7 ± 2.5	–

*Note*: *b*[*IC95*] is the coefficient of linear regression estimated with LMMs. See Table S3 for more details on LMMs.

* Bold values indicates significant test *p* < 0.001.

**FIGURE 2:**
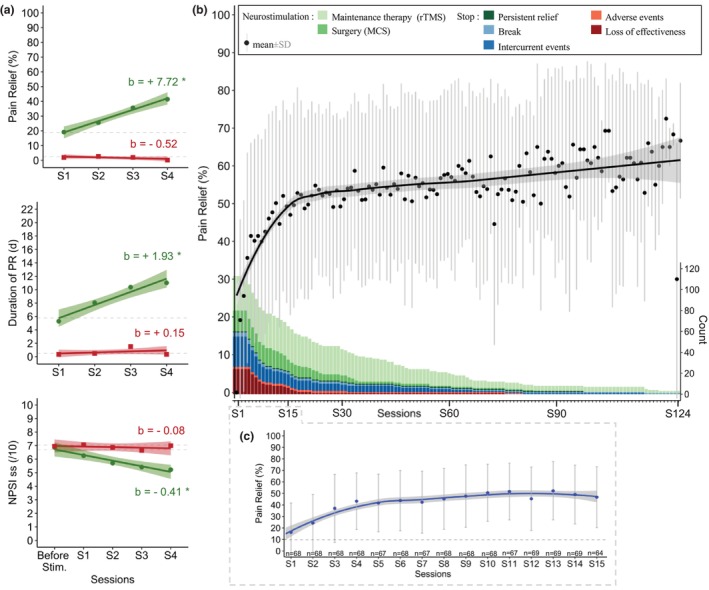
(a) Time course of outcomes (PR, DPR and ssNPSI) during the test period in the whole population according to status after the 4th session (responders: Green dot, and non‐responders: Red square). Regression lines (with 95% confidence intervals) and coefficients (b) obtained from linear mixed models (including a random intercept for each patient) with sessions as repeated measures factor. *indicates significant test for regression coefficients (*p* < 0.05). Note that responders (PR≥10%, *n* = 113) and non‐responders (PR < 10%, *n* = 80) did not differ at baseline in the ssNPSI (LMMs results; 6.69[6.14;7.25] vs. 7.04[6.52;7.57], *p* = 0.38) (b) Time course of percentage of pain relief over 124 sessions in responders (*n* = 113) and sample size of responders per session. The black line indicates smooth conditional means (with 95% confidence interval) of PR over 124 sessions and black dots are averages (±sd on grey) for each session. The sample size progressively decreases over time and the reasons for session discontinuation are detailed with colour bars. Note a progressive increase of PR during the first year (first 15th sessions) which may be due to the disappearance of ephemeral responders (patients who discontinued rTMS due to loss of efficacy) or to a real cumulative effect. (c) Time course of percentage of pain relief in responders who had received at least 15 sessions (*n* = 69). The blue line indicates smooth conditional means (with 95% confidence interval) of PR over the first 15 sessions and blue dots are averages (±sd on grey) for each session. Note a cumulative effect, reaching a saturation after the 6th session, at around 50% PR.

In responders (*n* = 113), a significant increase in the PR and the DPR were observed in LMMs, between the first and the fourth session (PR: *b* = 7.72[5.58;9.85] and DPR: *b* = 1.93[1.26;2.61], Figure 2a and Table 2). We observed a decrease in both ssNPSI (*b* = −0.41[−0.59; −0.24], Figure 2a) and gNPSI (*b* = −1.25[−2.08; −0.41], Table 2) between the baseline and the fourth session. In non‐responders (*n* = 80), no significant improvement was found for any pain assessments, as compared to the reference time (Figure 2a). A significant difference was found in the regression coefficients of variables discriminating positive responders from non‐responders (ssNPSI: *p* = 0.01, Figure 2a and gNPSI: *p* = 8.6e^−06^). See Table S3 for more details on LMMs.

### 3.3 Status change

After the test period (Figure 1a), 21% (*n* = 24) of responders discontinued the rTMS session due to loss of efficacy and 71% (*n* = 17) of them stopped before the 15th session, on average at the 7th session (7.1 ± 2.4) and were qualified as ‘ephemeral responders’. Conversely, six patients who were initially non‐responders (PR < 10%) at the 4th session became responders after the 6th rTMS session on average (5.8 ± 1.6) and were qualified as ‘late responders’. See Table S4 and Figure S2 for further information.

### 3.4 Long‐term maintenance

After the test period, responders adapted their frequency of iterative sessions to the duration of their pain relief and the interval between the 14th and 15th session was 24.8 (±11.8) days on average (see Figure S3). The average follow‐up length for responders was 2.2 years (±2.5) with a maximum follow‐up of 10 years and 4 months. The average number of sessions was 32.0 (±31.8) per patient with a maximum of 125 sessions (Figure 2b). In responders who have received at least 15 sessions (*n* = 69), iterative rTMS sessions induced a cumulative effect on the percentage of PR, reaching a saturation after the 6th session, at around 50% PR and which was maintained for more than 100 sessions in seven patients (Figure 2c). All reasons combined (from loss of efficacy to eMCS surgery), 33% of responders (37/112) discontinued rTMS sessions before the 15th session, 57% (61/107) before the 30th session and 91% (77/85) before the 90th session (reasons were detailed in Figures 1a and 2b. and see Table S5). Beyond the 30th session, the main reasons for discontinuation were intercurrent event or surgery.

### 3.5 rTMS and eMCS

**FIGURE 3:**
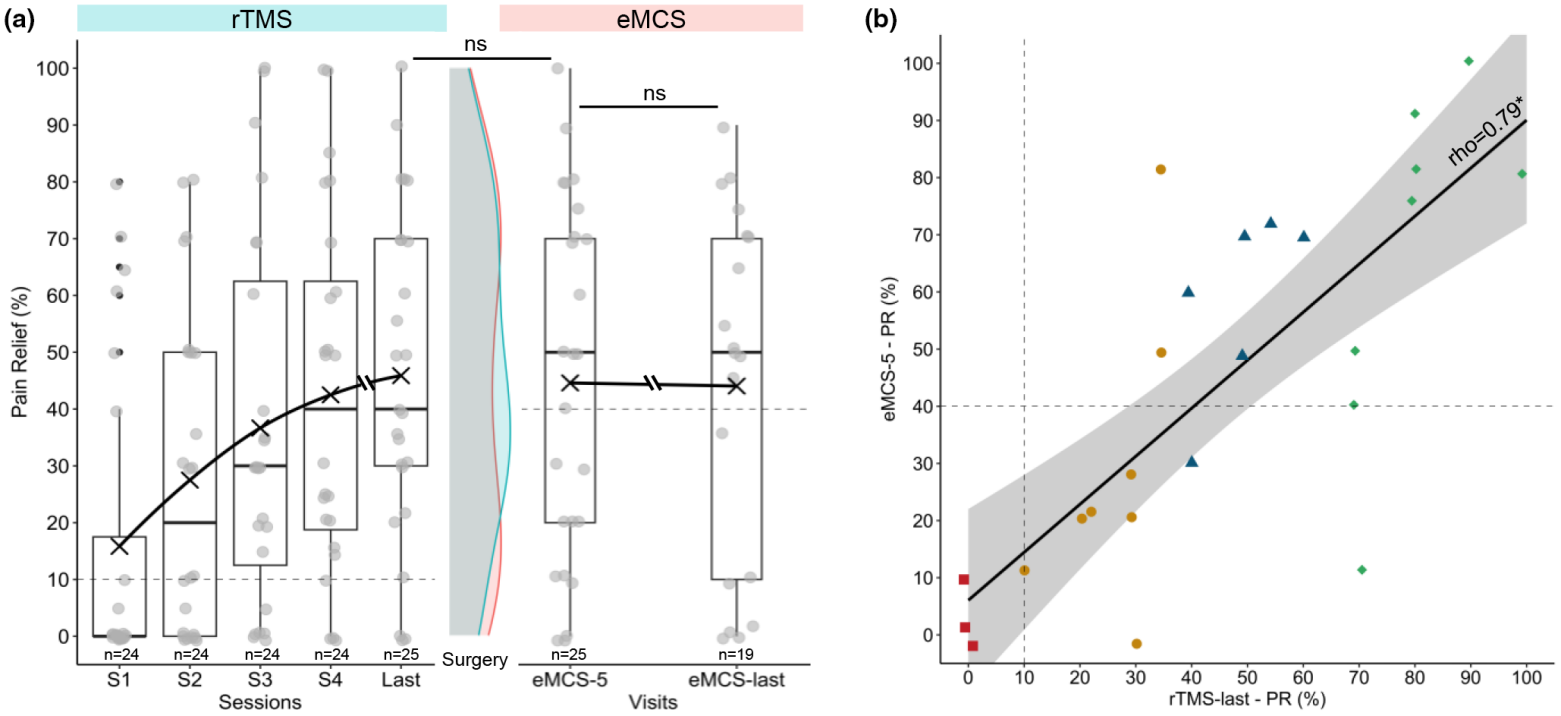
(a) Time course of percentage of pain relief during rTMS sessions and after eMCS. Individual dots represent raw data. The black cross represents the mean of each time, with a line connecting them. In the middle, comparison of density diagram between the last rTMS session (*rTMS‐last*, blue) and first eMCS evaluation (*eMCS‐5*, pink), and no significant difference was observed. The difference in patient numbers between the two evaluation periods for eMCS efficacy is due to six patients preferring thetaburst stimulation instead of classical stimulation. (b) Association between eMCS efficacy and rTMS efficacy. Simple linear regression model (with 95% confidence interval) between the percentage of pain relief at the first evaluation after surgery (*eMCS‐5*) and at the last rTMS session (Spearman test: rho = 0.79, *p* = 2.18e^−06^). Various colours/shapes of dots show how patients were categorized based on their final rTMS pain relief: 10% (*n* = 3) Failure (0%–9%, red square), 32% (*n* = 8) Poor result (10%–39%, yellow dot), 24% (*n* = 6) Good result (40%–69%, blue triangle), 32% (*n* = 8) Excellent result (≥70%, green diamond). Note that 6 patients reported a better PR with rTMS, 9 patients reported no difference and 10 patients reported a better PR with eMCS.

25 patients underwent eMCS after an average of 22.5 (±18.3 [min 4; max 84]) rTMS sessions, corresponding individually to 1.5 ± 1.6 years of rTMS treatment before the eMCS surgery (Table 3). Among them, 22 patients were rTMS responders. At the first evaluation post‐surgery (at 5.1 ± 1.4 months after surgery, *eMCS‐5*), analgesic effect of eMCS was rated as excellent in 36% (*n* = 9) of cases, good in 20% (*n* = 5), poor in 32% (*n* = 8) and failure in 12% (*n* = 3) (Table 3). Pain relief reported at the last available visit (*eMCS*‐*last*, 38.2 ± 23.7 months) was significantly correlated (rho = 0.51, *p* = 0.03) with the first evaluation (*eMCS*‐*5*), and no difference in efficacy was found (*eMCS‐5*: 44.6 ± 31.6 vs. *eMCS*‐*last*: 44.1 ± 31.3, *V* = 63, *p* = 0.82, Figure 3a). Pain relief at the last rTMS session and *eMCS‐5* was significantly correlated (Spearman test: rho = 0.79, *p* = 2.18e^−06^, Figure 3b) and no difference was found between the efficacy of rTMS and eMCS (45.9 ± 29.1 vs. 44.6 ± 31.6, *V* = 88, *p* = 0.93, Figure 3a).

**TABLE 3:** Characteristics and outcomes of the 25 patients who received eMCS.

	Variables	Values
**Baseline**	Age, years (mean ± SD)	53.9 ± 12.0
	Male sex, % (*n*)	48.0 (12)
	Pain duration, years (mean ± SD)	8.8 ± 6.9
	Pain laterality, % (*n*)	
	Right	44.0 (11)
	Left	56.0 (14)
	Pain topography, % (*n*)	
	Hemibody	60.0 (15)
	Lower limb	20.0 (5)
	Upper limb	8.0 (2)
	Face/neck	4.0 (1)
	Trunk	8.0 (2)
	Level of lesion, % (*n*)	
	Central	88.0 (22)
	Spinal cord	31.8 (7)
	Brainstem	22.7 (5)
	Thalamus	18.2 (4)
	Lenticular	4.5 (1)
	Cortex	18.2 (4)
	Others	4.5 (1)
	Peripheral	12.0 (3)
	Cranial	33.3 (1)
	Radicular	33.3 (1)
	Plexus	33.3 (1)
**rTMS treatment**	rTMS follow‐up time, years (mean ± SD)	1.5 ± 1.6
	Number of received sessions, session (mean ± SD)	22.5 ± 18.3
	Duration of PR at last rTMS session, *d* (mean ± SD)	13.3 ± 9.9
	Pain relief after rTMS at	
	Session 1, % (mean ± SD)	15.8 ± 27.5
	Session 2, % (mean ± SD)	27.5 ± 28.5
	Session 3, % (mean ± SD)	36.7 ± 32.8
	Session 4, % (mean ± SD)	42.5 ± 31.6
	Last session, % (mean ± SD)	45.9 ± 29.1
	Categorization after the last rTMS, % (*n*)	
	Non‐responder (0–10)	12.0 (3)
	Poor result (10–39)	32.0 (8)
	Good result (40–69)	24.0 (6)
	Excellent result (70–100)	32.0 (8)
**eMCS surgery**	Delay between last rTMS and surgery, *m* (mean ± SD)	4.8 ± 8.0
	Delay between surgery and first evaluation, *m* (mean ± SD)	5.1 ± 1.4
	Delay between surgery and last evaluation, *m* (mean ± SD)	38.2 ± 23.7
	Pain relief thanks to eMCS at	
	First evaluation, % (mean ± SD)	44.6 ± 31.6
	Last evaluation, % (mean ± SD)	44.1 ± 31.3
	Categorization after eMCS‐5, % (*n*)	
	Non‐responder (0–10)	12.0 (3)
	Poor result (10–39)	32.0 (8)
	Good result (40–69)	20.0 (5)
	Excellent result (70–100)	36.0 (9)

### 3.6 Etiological influence on efficacy

**FIGURE 4:**
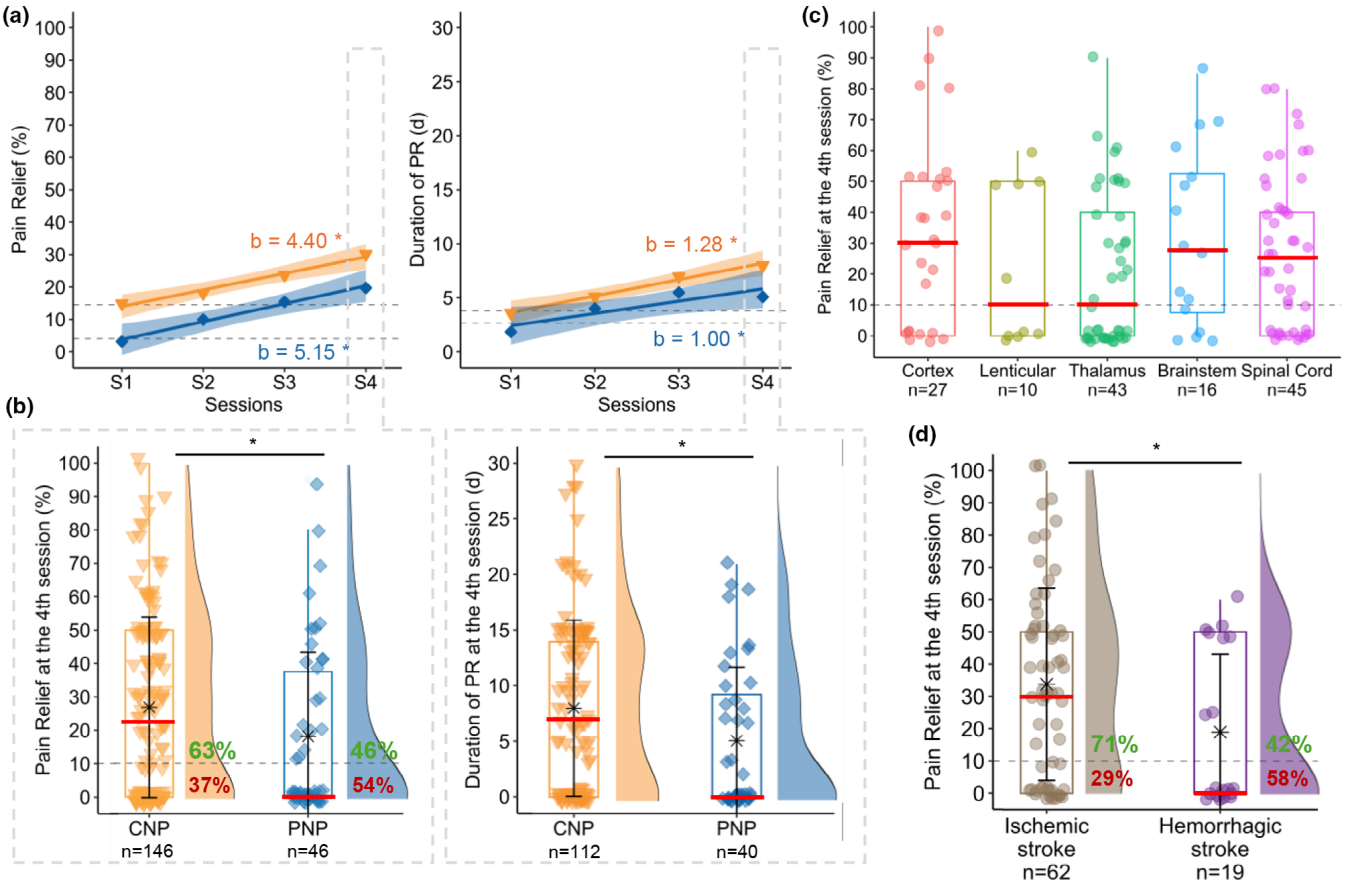
(a) Time course of percentage and duration of pain relief during the test period in the whole population according to aetiology (CNP: Central neuropathic pain in orange triangle, PNP: Peripheral neuropathic pain in blue diamond). Regression lines (with 95% confidence intervals) and coefficients (b) obtained from linear mixed models (including a random intercept for each patient) with sessions as repeated measures factor. *indicates a significant test for regression coefficients (*p* < 0.05). No significant difference was found in the regression coefficients of PR and DPR between CNP and PNP. (b) Comparison of percentage and duration of pain relief at the 4th session between CNP (on left) and PNP (on right). Boxplots and density plots show the distribution of individual data within each group. Means ± standard deviations are depicted as stars with error bars. Medians are represented by red lines. On density diagrams, we can see the percentage of responders (≥10% PR) in CNP (=63%) and PNP (=46%). *indicates a significant difference between the 2 groups (*p* < 0.05). (c) Distribution of percentage of PR at the 4th session according to five levels of lesion (cortex, lenticular, thalamus, brainstem, spinal cord) in patients with central NP. (d) Distribution of percentage of PR at the 4th session according to the type of the stroke (i.e. ischemic and hemorrhagic) in patients with central NP. *indicates a significant difference between the 2 groups (*p* < 0.05).

No significant difference was found in the regression coefficients, estimated with LMMs, of PR and DPR between CNP and PNP (please see Figure 4a and Table S6 for further information). The proportion of responders after the test period was 63%(*n* = 92/146) in central NP and 46%(*n* = 21/46) in peripheral NP (Figure 4b). Patients with CNP have a higher probability to experience pain relief than patients with PNP (OR = 2.03[1.04;4.00], *p* = 0.04) and the duration of PR after the 4th session was significantly higher for CNP patients than for PNP (8.0 ± 7.9 vs. 5.1 ± 6.6, *p* = 0.03, Figure 4b). In responders (*n* = 113), we found no difference between CNP and PNP neither for the percentage of PR (42.6 ± 22.0 vs. 39.9 ± 22.7, *t* = 0.49, *p* = 0.63), nor for the duration (11.1 ± 7.4 vs. 10.5 ± 5.9, *W* = 788.5, *p* = 0.74) at the 4th session (see Table S7 and Figure S4). Among patients with CNP, the sample size was large enough to describe five groups of lesions (Figure 4c). At the 4th session, patients with ischemic stroke (*n* = 62) were more likely to respond to rTMS than patients with hemorrhagic stroke (*n* = 19; OR = 3.36[1.17, 10.05], *p* = 0.03, Figure 4d). Concerning the other etiologies identified, the percentage of responders was 100% for patients with multiple sclerosis (*n* = 9), 75% for trigeminal neuralgia (*n* = 8), 63% for patients whose pain was of oncological origin (*n* = 8) and 33% for sensory polyneuropathies (*n* = 6) such as diabetes or Sjögren's syndrome.

## 4 DISCUSSION

The study suggests the potential clinical relevance of robotized and neuronavigated rTMS in patients with neuropathic pain, with evidence pointing to a long‐term maintenance of analgesia in certain cases. A proportion of 25%–59% of patients with drug‐refractory NP experienced clinical benefits with iterative rTMS sessions and some of them had prolonged pain relief for several years. A similar pattern of effects was evident for the NPSI (sub‐scores and global), which decreased significantly among responders between the baseline and the fourth session. This finding highlights the utility of the NPSI, particularly the sub‐score, in assessing pain improvement and differentiating treatment response. These results are encouraging, especially as they concern patients suffering from severe, chronic and pharmaco‐resistant neuropathic pain. In comparison, first‐line medications for neuropathic pain, such as gabapentinoids or tricyclic antidepressants, often provide moderate pain relief in around 30%–50% of patients, though their long‐term efficacy can be limited by tolerance or side effects (Finnerup et al., 2015; Moisset, Bouhassira, et al., 2020). rTMS presents a favourable safety profile, with no serious adverse events reported. Mild side effects, such as headaches or transient pain exacerbation due to head posture during the sessions, were rare and consistent with existing literature (Gatzinsky et al., 2021; Loo et al., 2008). The absence of an intensive induction phase and the use of a robotized arm to ensure that the probe is positioned optimally on the head without excessive pressure may have contributed to the low rate of side effects in our cohort.

Our results indicate that the time to reach a plateau in pain relief (around 50%) may be longer than previously reported (Quesada et al., 2018), particularly for patients with initially low responses. Interestingly, patients with 10–29% initial pain relief can still achieve moderate relief (30%–49%) after 15 sessions of rTMS. This suggests the need to reconsider the current clinical approach with the aim to avoid premature exclusion of potential responders: Either the number of sessions before determining responsiveness is extended, or a more permissive evaluation is adopted, considering patients with only a 10% improvement after four sessions as potential future responders. These conclusions align with evolving recommendations, which have transitioned from one single session predictor of efficacy in 2011 (André‐Obadia et al., 2011) to five sessions in recent guidelines (Lefaucheur & Nguyen, 2019). Hence, we propose the amount of 5 rTMS sessions as a minimum to assess treatment efficacy.

The efficacy of neurosurgery could be positively predicted by those of iterative rTMS sessions, consistent with previous findings using one single predictive session (André‐Obadia et al., 2014; Lefaucheur et al., 2011). All patients with a response to rTMS had a similar ±10%–20% pain relief with eMCS, with an excellent linear correlation between the two techniques. Such filtering of patients with rTMS sessions has totally changed the way to recommend eMCS procedure to patients since in our recent experience, we no longer observed patients undergoing a no‐result neurosurgery.

In line with data from the literature, our cohort demonstrates a high variability in response, both in the responder rate and in the magnitude of response. Understanding why some patients respond positively to rTMS while others do not have been a central debate among rTMS experts for years. Current strategies to predict the response to rTMS are primarily based on assessing M1 stimulation patterns, cortical networks and rhythms (Ciampi de Andrade & García‐Larrea, 2023). In our study, we aimed to explain this variability by examining factors such as lesion level and pain aetiology. Most of our patients had central NP, probably due to a historical recruitment bias in our pain centre. Following the recent trial by Attal and colleagues (Attal et al., 2021), we also proposed to patients with peripheral NP, the same procedure that had been routinely organized for CNP. This extension to patients with peripheral NP allows a direct comparison with a respectable sample size. Success rates after 4 rTMS sessions were lower for peripheral (46% responders) as compared to central NP (63% responders). These results are consistent with a meta‐analysis indicating differential analgesic effects based on neuroanatomical origins of the NP pathophysiology with a more effective response to treatment observed in spinal or supraspinal lesions than after nerve root or peripheral nerve lesions (Leung et al., 2009). The present results may also be influenced by the aetiology in the peripheral NP group with only a few diabetic patients, and conversely a large proportion of trigeminal pain in our cohort, as compared to previous literature. Regarding the aetiology in patients with central neuropathic pain, we observed a predisposition for a better analgesic effect in patients with ischemic as compared to hemorrhagic strokes, confirming a previous statistical trend (Zhao et al., 2021). Both the extent of the lesion and the over‐representation of lenticular hematoma could explain this difference in favour of ischemia. A consecutive explanation could be that hemorrhagic strokes, by affecting the white matter, could disrupt the connections linking the primary motor cortex to other deep structures such as thalamus or basal ganglia (Mandonnet et al., 2024). Except these differences, predictive biomarkers remain limited at the time. In that respect, a special mention should be made for the subgroup of patients with thalamic stroke which is the largest one. Lesions are almost all concentrated in a very small volume that is reproducible from one patient to the other because of vasculature specificities. Despite this relatively reproducible lesion, we failed to identify a common profile of response for this subgroup, the proportion of responders/non‐responders being almost identical to those of the cohort. Differences within this group may relate to impairments in lemniscal and/or spinothalamic circuits, but we cannot conclude on this possible marker. Integrating clinical variables into predictive models could offer a valuable approach, not only by refining therapeutic strategies but also by being easier to implement in routine practice. For instance, a recent study proposed an algorithm for peripheral neuropathic pain, incorporating clinical markers such as depressive symptoms, pain magnification and a variable related to the pain body area (Attal et al., 2024). Further research in this direction would be worthwhile.

We acknowledge several limitations in our study. First, it lacked a placebo control, typical of real‐world settings for clinic. Nonetheless, the procedure presented in this study replicated the same conditions as in a clinical trial validating the efficacy of rTMS against placebo (Quesada et al., 2020). While data collection followed standardized procedures, the retrospective nature of the study limited data availability. Other longitudinal measures may be important to assess the efficacy and added value of rTMS in patients with multiple comorbidities such as depression (Dworkin et al., 2005). Although the percentage of pain relief was chosen as the primary outcome in this study due to its relevance in tracking pain improvement over time, guidelines recommend using the evolution of the VAS as the main criterion for pain assessment (Dworkin et al., 2005). This methodological choice may limit the comparability of our results with studies that primarily use the VAS. Furthermore, the use of self‐report changes in pain, rather than absolute pain reports, may introduce recall bias. However, the consistency observed between the percentage of pain relief and the NPSI sub‐scores mitigates this limitation. To address these concerns in future studies, we recommend combining traditional pain scales (such as VAS) with self‐reported improvement scores, like the percentage of pain relief or the Patient Global Impression of Change scale (PGIC), both of which have been successfully used in previous rTMS trials (Attal et al., 2021; Quesada et al., 2020). After the test period, treatments against pain have been used transiently and/or discontinued possibly influencing the follow‐up. The strict French context of the study may limit generalizability in other countries. Data were collected between 2010 and 2022. During this period, health care has been discontinued for several patients due to the global health crisis caused by COVID‐19 and this may be a confound to the results. Finally, over the 12‐year study period, evolving knowledge, risks, benefits and technical refinements may have impacted practice and outcomes.

## 5 CONCLUSIONS

Our findings indicate good acceptability, safety and stable effects, even over long periods, with potential cumulative carry‐over effects in a highly inclusive population. Following test period, central neuropathic pain may show higher success rates compared to peripheral NP and ischemic lesions may have a better prognosis than hematoma. This observational study provides information on how rTMS can be routinely used with clinical settings in a pain centre before orienting patients to a surgical eMCS.

## AUTHOR CONTRIBUTIONS

This study was designed by J.T., R.P., C.F. and C.Q. The data collection was performed by J.T., C.Q., F.V., C.C. and N.O. The data were analysed by J.T., and the results were critically examined by all authors. J.T., R.P. and C.F. contributed to the redaction of the manuscript. All authors have approved the final version of the manuscript and agree to be accountable for all aspects of the work.

## CONFLICT OF INTEREST STATEMENT

The authors have no conflicts of interest to declare.

## Supporting information


**Data S1:** Supplementary Information.
