# Supplementary material for: Effects of multiple transcranial magnetic stimulation sessions on pain relief in patients with chronic neuropathic pain: A French cohort study in real‐world clinical practice
Source: Eur J Pain. 2024 Dec 10;29(1):e4763. doi: 10.1002/ejp.4763 (PMC11629460; doi:10.1002/ejp.4763)
Supplement: Supplementary file 1 — Data S1: Supplementary Information. [file EJP-29-0-s001.docx]

***SUPPLEMENTARY***

**Sample characteristics**


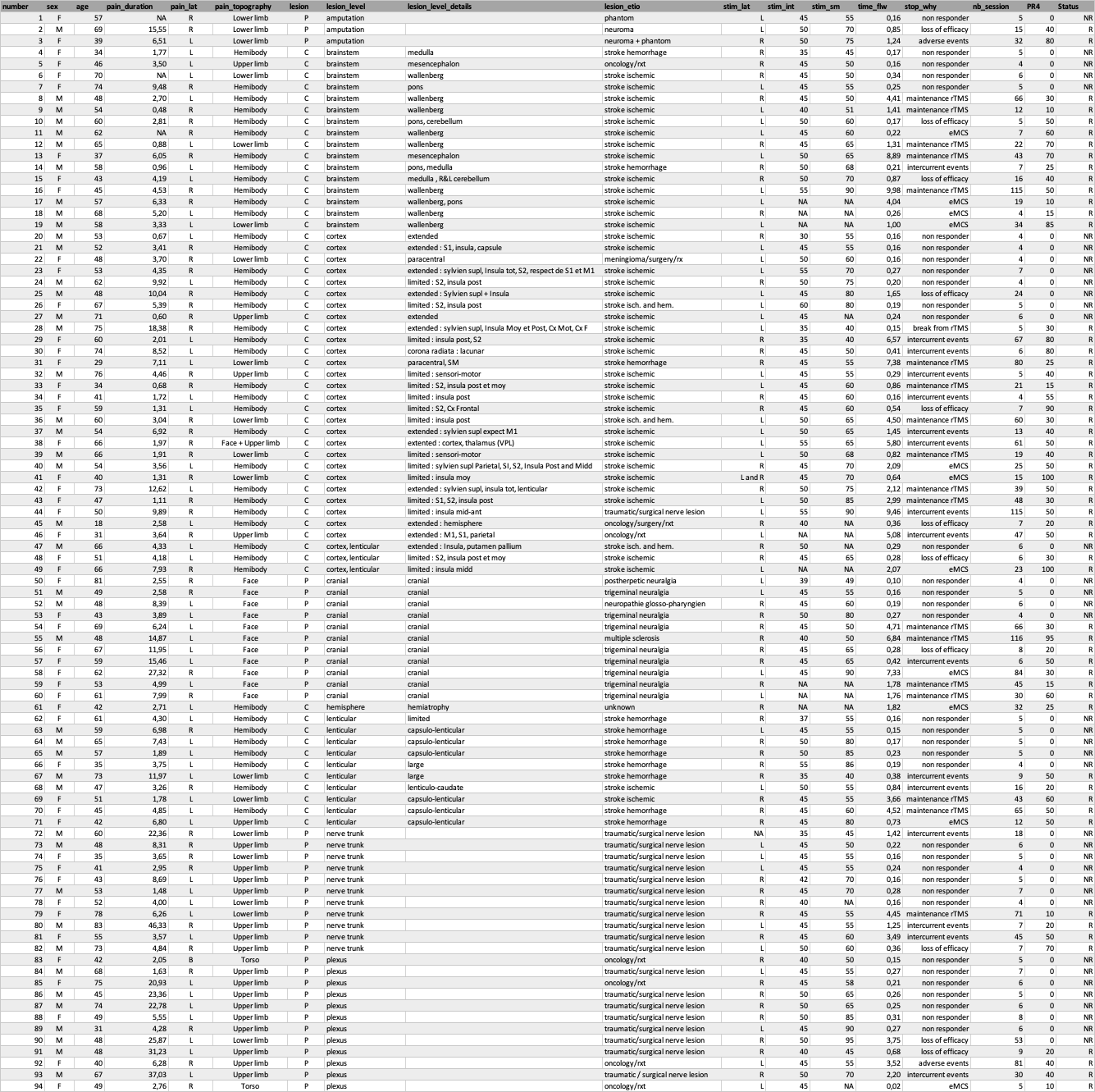
Table S1: Patient’s clinical characteristics and results of rTMS-treatment

*
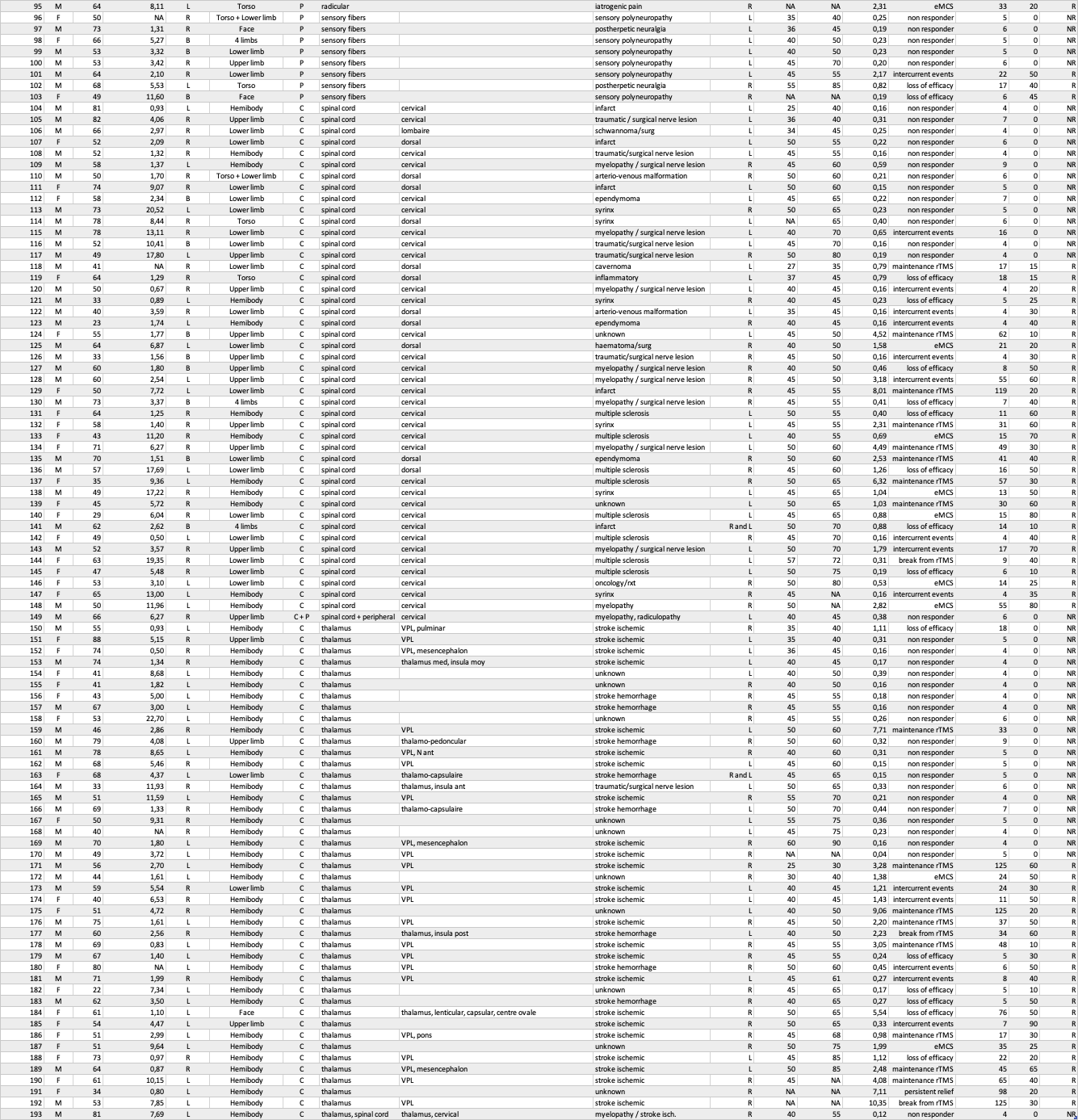
 M: male, F: female, L: left, R: right, C: central NP, P: peripheral NP, VPL: ventro-postero-lateral nucleus, R: responders, NR: non-responders*

**Categorization and main outcomes over time**

|  | PR 10-29%  N=32 | PR 30-49%  N=32 | Tests |
| --- | --- | --- | --- |
| Time follow, y (mean ± SD) | 2.0 ± 2.5 | 2.3 ± 2.6 | W=540,5, p=0.71 (n=64) |
| Number of session, (mean ± SD) | 28.6 ± 33.1 | 32.8 ± 31.4 | W=541, p=0.70 (n=64) |
| Discontinuation due to lack of efficacy, % (n) | 28.1 (9) | 21.9 (7) | X=0.33, p=0.56 (n=64) |
| PR 15th session, % (mean ± SD) | 31.9 ± 22.4 | 46.5 ± 27.5 | W=100.5, p=0.08 (n=35) |
| PR 30th session, % (mean ± SD) | 35.9 ± 17.9 | 42.7 ± 20.1 | W=44.5, p=0.30 (n=22) |

Table S2: Effectiveness of rTMS according to level of pain relief (PR) reported at the 4th session. Please note no statistical difference between patients reporting a PR between 10 and 29% and those reporting a PR between 30 and 49% on the variables analyzed.

Figure S1: Evolution of the percentage of pain relief according to the level of response reported at the 4th session. The number of patients analyzed per session decreases over time due to rTMS discontinuation, including reasons related to rTMS, with no statistically significant difference between the 10-29% response group and the 30-49% response group.

Table S3: Detailed results of linear mixed models (LMMs) and comparison of coefficients and reference time between responders and non-responders. * indicates significant test (p<0.05)

|  |  | **Whole population**  **N=193** | **Responders**  **N=113** | **Non responders**  **N=80** | **R vs NR** | |
| --- | --- | --- | --- | --- | --- | --- |
| **Pain relief (%)** | | | | | | |
| mean | S1 | 11.89 [8.90;14.89] | 18.89 [14.35;23.43] | 2.53 [0.78;4.28] | **p=3.56e^-08^** | |
|  | Slope, b[IC95] | **4.55 [3.09;6.01] *** | **7.72 [5.58;9.85] *** | -0.52 [-1.43;0.40] | **p=1.51e^-08^** | |
| variability | Intercept | 15.99 [15.20;17.57] | 17.99 [13.22;22.58] | 4.23 [0;6.48] |  | |
|  | Slope | 6.67 [4.94;8.25] | 7.11 [4.01;9.61] | 1.62 [0;3.28] |  |  |
|  | Correlation | -0.09 [-0.35;0.29] | **-0.53 [-0,74;-0,11] *** | -1 [-1;1] |  |  |
| ICC (%) | | 53 [42;62] | 49 [31;62] | 25 [4;48] |  |  |
| **Duration of pain relief (d)** | | | | | | |
| mean | S1 | 3.48 [2.54;4.43] | 5.77 [4.27;7.26] | - | **p=1.60e^-08^** | |
|  | Slope, b[IC95] | **1.21 [0.77;1.64] *** | **1.93 [1.26;2.61] *** | - | **p=4.11e^-05^** | |
| variability | Intercept | 4.86 [3.91;5.80] | 5.86 [4.36;7.33] | - |  | |
|  | Slope | 1.71 [1.06;2.25] | 2.17 [1.17;2.97] | - |  |  |
|  | Correlation | -0.10 [-0.39;-0.41] | -0,60 [-0.79;-0.23] | - |  |  |
| ICC (%) | | 50 [37;60] | 52 [35;64] | - |  |  |
| **Global NPSI (/100)** | | | | | | |
| mean | Baseline | 37.67 [34.92;40.42] | 38.32 [34.40;42.25] | 36.89 [33.16;40.63] | p=0.63 | |
|  | Slope, b[IC95] | 0.07 [-0.62;0.76] | **-1.25 [-2.08;-0.41] *** | **1.84 [0.81;2.87] *** | **p=8.6e^-06^** | |
| variability | Intercept | 16.70 [14.62;18.97] | 18.16 [15.25;21.44] | 14.58 [11.77;17.79] |  | |
|  | Slope | 3.20 [2.48;3.90] | 2.57 [1.40;3.56] | 3.09 [2.00;4.14] |  |  |
|  | Correlation | 0.12 [-0.12;0.40] | 0.07 [-0.27;0.57] | 0.33 [-0.07;0.79] |  |  |
| ICC (%) | | 77 [72;82] | 79 [72;85] | 73 [63;81] |  |  |
| **Sub-score NPSI (/10)** | | | | | |  |
| mean | Baseline | 6.85 [6.46;7.23] | 6.69 [6.14;7.25] | 7.04 [6.52;7.57] | p=0.38 |  |
|  | Slope, b[IC95] | **-0.28 [-.41;-0.15] *** | **-0.41 [-0.59;-0.24] *** | -0.08 [-0.27;0.09] | **p=0.0123** |  |
| variability | Intercept | 1.80 [1.47;2.14] | 1.93 [1.47;2.44] | 1.61 [1.16;2.10] |  |  |
|  | Slope | 0.51 [0.37;0.65] | 0.51 [0.31;0.70] | 0.46 [0.23;0.67] |  |  |
|  | Correlation | -0.09 [-0.37;0.28] | -0.10 [-0.45;0.44] | -0.20 [-0.56;0.51] |  |  |
| ICC (%) | | 62 [50;70] | 62 [47;73] | 62 [45;75] |  |  |

**Status change**

Table S4: Description at baseline of patients according to status at the 15^th^ session. *R: responders, NR: non-responders*

|  |  | Ephemeral R  N = 17 | NR  N = 74 | Late R  N = 6 | R  N = 96 |
| --- | --- | --- | --- | --- | --- |
| Male sex, % (n) |  | 58.8 (10) | 56.8 (42) | 100 (6) | 45.8 (44) |
| Age, y (mean±SD) |  | 53.9 ± 16.3 | 58.3 ± 14.0 | 55.8 ± 12.3 | 55.1 ± 12.9 |
| Level of lesion, % (n) | Central | 76.5 (13) | 67.6 (50) | 66.7 (4) | 82.3 (79) |
|  | Peripheral | 23.5 (4) | 31.1 (23) | 33.3 (2) | 17.7 (17) |
|  | Mixt | 0 (0) | 1.3 (1) | 0 (0) | 0 (0) |
| Pain laterality, % (n) | Left | 52.9 (9) | 47.3 (35) | 33.3 (2) | 51.0 (49) |
|  | Right | 23.5 (4) | 45.9 (34) | 66.7 (4) | 45.8 (44) |
|  | Bilat | 23.5 (4) | 6.8 (5) | 0 (0) | 3.1 (3) |
| Pain duration, y (mean±SD) |  | 5.8 ± 7.4 | 6.0 ± 5.6 | 12.5 ± 10.1 | 6.5 ± 7.3 |
| NRS, /10 (mean±SD) |  | 5.6 ± 2.3 | 6.8 ± 1.9 | 4.2 ± 2.1 | 6.1 ± 2.1 |
| Global NPSI, /100 (mean±SD) |  | 34.8 ± 15.1 | 37.1 ± 18.0 | 26.2 ± 9.3 | 37.3 ± 19.0 |

Figure S2: Time course of pain relief (PR) based on patient status at the 15th session (± Standard Error of the Mean, SEM). Dotted lines represent the PR progression of non-responders and ephemeral responders after the 4th session, as the number of patients analyzed decrease after this landmark due to lack or loss of efficacy. The number of patients analyzed decrease also for responders due to discontinuation for reasons unrelated to efficacy (e.g., implantation or intercurrent events).

**Long-term maintenance**

Figure S3: Evolution of the interval between each session (in day) in whole population (black dots: mean, grey bars: SEM). For exemple; “session=2” refers to the time between the first and second session.

Table S5: Description of responders at the fourth session (n=113), and their rTMS follow-up depending on their outcome at the end point.

|  | **Neurostimulation (N=53)** | | **Stop (N=60)** | | | | |
| --- | --- | --- | --- | --- | --- | --- | --- |
|  | **Noninvasive**  **(rTMS)** | **Invasive**  **(eMCS)** | **Persistent relief** | **Break from rTMS session** | **Intercurrent events** | **Adverse events** | **Loss of effectiveness** |
| Ntot =113 | n=33 | n=20 | n=1 | n=4 | n=29 | n=2 | n=24 |
| **Age**, y (mean ± SD) | 54.9 ± 12.6 | 52.4 ± 10.4 | 34.5 | 63.0 ± 9.2 | 56.3 ± 14.9 | 39.8 ± 0.5 | 56.3 ± 15.1 |
| **Male sex**, % (n) | 36.4 (12) | 50.0 (10) | 0.0 (0) | 75.0 (3) | 55.2 (16) | 0.0 (0) | 54.2 (13) |
| **Level of lesion**, % (n) Central | 84.8 (28) | 85.0 (17) | 100.0 (1) | 100.0 (4) | 82.8 (24) | 0.0 (0) | 75.0 (18) |
| Peripheral | 15.2 (5) | 15.0 (3) | 0.0 (0) | 0.0 (0) | 17.2 (5) | 100.0 (2) | 25.0 (6) |
| **Pain laterality**, % (n) Bilat | 6.1 (2) | 0.0 (0) | 0.0 (0) | 0.0 (0) | 3.4 (1) | 0.0 (0) | 16.7 (4) |
| Right | 42.4 (14) | 45.0 (9) | 0.0 (0) | 75.0 (3) | 48.3 (14) | 50.0 (1) | 29.2 (7) |
| Left | 51.5 (17) | 55.0 (11) | 100.0 (1) | 25.0 (1) | 48.3 (14) | 50.0 (1) | 54.2 (13) |
| **Pain duration**, y (mean ± SD) | 4.6 ± 3.7 | 7.5 ± 6.3 | 0.8 | 12.0 ± 8.2 | 7.3 ± 10.5 | 6.4 ± 0.2 | 6.0 ± 7.1 |
| **Pain intensity** (NRS), /10 (mean ± SD) | 6.2 ± 1.8 | 6.2 ± 2.0 | 6.0 | 5.0 ± 1.6 | 6.1 ± 2.5 | 6.0 ± 0.0 | 5.8 ± 2.5 |
| **Global score** NPSI, /100 (mean ± SD) | 40.6 ± 17.5 | 42.4 ± 15.1 | 66.0 | 37.5 ± 5.0 | 30.0 ± 21.6 | 26.0 ± 28.3 | 35.5 ± 15.7 |
| **Level of responders after 4** **sessions**, % (n) Poor | 51.5 (17) | 45.0 (9) | 100.0 (1) | 50.0 (2) | 27.6 (8) | 0.0 (0) | 45.8 (11) |
| Good | 39.4 (13) | 25.0 (5) | 0.0 (0) | 50.0 (2) | 58.6 (17) | 50.0 (1) | 45.8 (11) |
| Excellent | 9.1 (3) | 30.0 (6) | 0.0 (0) | 0.0 (0) | 13.8 (4) | 50.0 (1) | 8.3 (2) |
| **rTMS follow-up time**, y (mean ± SD) | 3.90 ± 2.6 | 1.7 ± 1.7 | 7.11 | 3.3 ± 4.8 | 1.7 ± 2.3 | 2.4 ± 1.6 | 0.7 ± 1.1 |
| **Received more than 15 sessions**, yes % (n) | 97.0 (32) | 70.0 (14) | 100.0 (1) | 50.0 (2) | 37.9 (11) | 100.0 (2) | 29.2 (7) |
| **Number of received sessions,** session (mean ± SD) | 55.4 ± 32.7 | 24.3 ± 18.7 | 98.0 | 43.3 ± 56.0 | 21.1 ± 26.1 | 56.5 ± 34.7 | 12.5 ± 14.4 |
| **PR at last rTMS session**, % (mean ± SD) | NA | 52.8 ± 25.1 | 80.0 | 37.5 ± 22.2 | 43.1 ± 28.0 | 60.0 ± 28.3 | 9.4 ± 17.8 |

**Variables influencing global pain relief and pain relief duration**

|  |  | **Central**  **N=146** | **Peripheral**  **N=46** | **CNP vs PNP** |
| --- | --- | --- | --- | --- |
| **Pain relief (%)** | | | | |
| mean | S1 | 14.46 [10.77;18.15] | 4.03 [0.24;7.82] | **p=0.003** |
|  | Slope | **4.40 [2.64;6.16]*** | **5.15 [2.53;7.77]*** | p=0.617 |
| variability | Intercept | 17.10 [13.44;20.72] | 7.99 [1.69;12.40] |  |
|  | Slope | 6.62 [4.33;8.60] | 6.96 [4.48;9.47] |  |
|  | Correlation | -0.11 | 0.08 |  |
| **Duration of pain relief (d)** | | | | |
| mean | S1 | 3.79 [2.65;4.92] | 2.66 [0.95;4.38] | p=0.293 |
|  | Slope | **1.28 [0.74;1.82]*** | **1.00 [0.30;1.71]*** | p=0.650 |
| variability | Intercept | 5.14 [4.02;6.24] | 4.04 [2.46;5.82] |  |
|  | Slope | 1.96 [1.26;2.56] | 0.62 [0.06;1.31] |  |
|  | Correlation | -0.23 | 1.00 |  |

Table S6: Detailed results of linear mixed models (LMMs) and comparison of coefficients and reference time between central and peripheral; during the four first sessions. * indicates significant test (p<0.05)

|  | Central NP | Peripheral NP | Tests |
| --- | --- | --- | --- |
| Pain relief at 4S, % (mean±SD) | 42.6±22.1 | 39.9±22.7 | t=0.49, p=0.63 |
| Duration of PR at 4S, d (mean±SD) | 11.1±7.4 | 10.5±5.9 | W=788.5, p=0.74 |
| Pain relief at 15S, % (mean±SD) | 45.9±24.6 | 52.1±34.2 | W=275, p=0.53 |
| Duration of PR at 15S, d (mean±SD) | 15.5±11.8 | 14.8±13.2 | W=362, p=0.70 |

Table S7: Comparison of the efficacy rTMS between CNP and PNP, in responders (n=112). Please note no difference on percentage nor duration of pain relief.


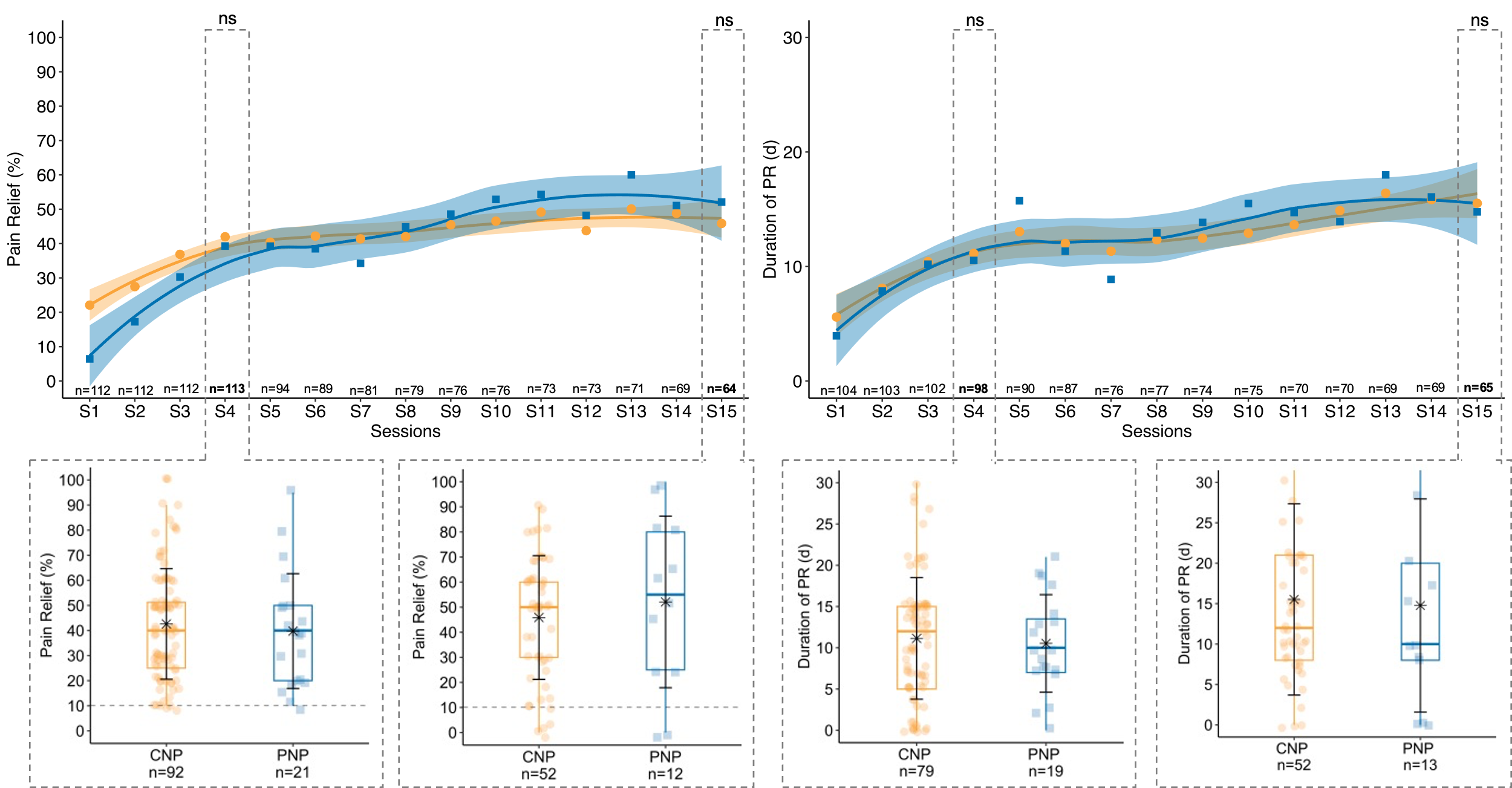


Figure S4: In responders, evolution of PR (on left) and DPR (on right) according to level lesion (orange dots = Central NP, blue squares= Peripheral NP). Comparison between two groups at 2 landmarks: 4th and 15th sessions. NS indicates no significant test (p>0.05). Please note the perfect match between the 2 groups on responders.
